# Spexin Levels Are Associated with Metabolic Syndrome Components

**DOI:** 10.1155/2018/1679690

**Published:** 2018-09-04

**Authors:** Nasser M. Al-Daghri, Amal Alenad, Hazim Al-Hazmi, Osama E. Amer, Syed Danish Hussain, Majed S. Alokail

**Affiliations:** Prince Mutaib Chair for Biomarkers of Osteoporosis, Biochemistry Department, College of Science, King Saud University, Riyadh 11451, Saudi Arabia

## Abstract

**Background:**

Spexin (SPX) is a novel peptide that is implicated in obesity and related energy homeostasis in animals and adult humans. Little is known about its role in adults' overall cardiometabolic health. The aim of the study was to determine whether circulating levels of spexin (SPX) is associated with components of metabolic syndrome (MetS).

**Methods:**

The present cross-sectional study included 124 participants (41 males and 83 females; aged 42.4 ± 10.3 y) (MetS group) and 136 (21 male and 115 females; aged 33.1 ± 8.7 y) (non-MetS group). SPX was measured using commercially available assays. Anthropometrics were measured, and fasting serum glucose levels as well as lipid profile were quantified routinely. MetS was screened according to common definitions.

**Results:**

SPX levels were significantly lower in participants with MetS vs. non-MetS (0.18 ng/ml (0.13–0.24) vs. 0.26 ng/ml (0.17–0.50); *p* < 0.001). In all MetS definitions used, SPX was significantly lower in the MetS group than the non-MetS group using the WHO definition after adjustment for age and BMI. Stratification according to sex revealed that SPX was associated with MetS only in women, and this significance was lost after adjustment for age and BMI.

**Conclusions:**

Lower circulating levels of SPX in adults are modestly associated with components of MetS and are sex-specific. Further studies are necessary to determine whether SPX is associated with harder outcomes such as atherosclerosis and diabetes in the general population.

## 1. Introduction

Spexin (SPX) is a novel peptide hormone initially identified using a computational method based on Markov model screening to identify novel biologically active peptides [1] which were later confirmed in microarray studies conducted in animals [2]. Subsequent tissue studies in humans showed that SPX was intensely expressed in normal human endocrine and epithelial tissues, indicating that SPX may be involved in physiological functions of endocrine and in several other tissues [3]. Furthermore, circulating SPX levels were observed to be low in T2DM patients and inversely related to blood glucose and lipids, suggesting that its potential role in glucose and lipid metabolism. In fish models, SPX is involved in various metabolic regulations, including satiety control [4, 5]. SPX levels were noted to be significantly lower in obese children but showed no associations with markers of insulin resistance (Kumar et al., 2016). This lack of association with glucose control and metabolism extends to adolescents (Hodges et al., 2017), and the link to obesity is further strengthened with the low SPX/high leptin observation, also among adolescents (Kumar et al., 2017). Results from pilot investigations in adults contradict findings observed in children and adolescents, with significant inverse associations between SPX and glycemic control as well as lipids and obesity [3, 6]. SPX as a peptide hormone is potentially important as a diagnostic and therapeutic target due to their wide range of physiological functions.

Metabolic syndrome (MetS) is a cluster of risk factors associated with cardiovascular disease and diabetes. Various groups including the World Health Organization (WHO) [7], European Group for the Study of Insulin Resistance [8], American Association of Clinical Endocrinologists [9], National Cholesterol Education Program-Adult Treatment Panel III (NCEP ATP III) [10], the International Diabetes Federation (IDF), and the American Heart Association (AHA) uniformly characterized MetS based on key cardiometabolic parameters [11] including obesity, elevated glucose, elevated blood pressure, hypertriglyceridemia, and low HDL cholesterol. The prevalence for MetS varies between countries, and it has been reported to be 28% in Saudi adults [12].

Given the emerging role of SPX in metabolism, the aim of this study was to further investigate the role of SPX in the development of MetS in adults with various clinical risk factors and whether circulating SPX correlates with the clinical parameters relative to MetS.

## 2. Methods

### 2.1. Subjects

A total of 260 (62 males and 198 females) participants were recruited from different public schools and health centers in collaboration with the Prince Mutaib Chair for Biomarkers on Osteoporosis (PMCO) at King Saud University (KSU), Riyadh, Saudi Arabia. The selection was made from an already existing master database [13]. Subjects were divided into two groups: those with MetS (*N* = 124; aged 42.4 ± 10.3 years) and those without MetS (non-MetS) (*N* = 136; aged 33.1 ± 8.7 years). Participants who were pregnant with acute conditions or complications including renal, hepatic, neurologic, and pulmonary diseases were excluded. The process of recruiting the subjects and the study performed followed the ethical principles of the Helsinki Declaration and was approved by the Ethics Committee of the College of Medicine in KSU, Riyadh, Saudi Arabia.

### 2.2. Clinical Assessment

Participating subjects were requested to return to their respective schools and primary health care centers (PHCCs) after an overnight fast for anthropometry and blood withdrawal. Anthropometry was measured and included height (to the nearest 0.5 cm) utilizing a standardized measuring tape in cm, weight (to the nearest 0.1 kg), and BMI (calculated as kg/m^2^). Resting blood pressure was measured twice by the standard procedure, and the average was recorded. Blood was transferred immediately to a nonheparinized tube for centrifugation.

### 2.3. Laboratory Parameters

The measurement of glucose levels and lipid profile of the fasting blood were performed using a chemical analyzer (Konelab, Vantaa, Finland). Circulating SPX measurements were performed using an enzyme-linked immunosorbent assay (ELISA) following the instruction of the manufacturer (Phoenix Pharmaceuticals Inc., Burlingame, CA) with a linear range of 0.11–1.07 ng/ml and intra-assay variation of <15%. Vitamin D (total 25(OH)) measurements were carried out using a COBAS e-411 automated analyzer (Roche Diagnostics, Indianapolis, IN, USA). All measurements were performed at PMCO, KSU, Riyadh, Saudi Arabia.

### 2.4. MetS Diagnosis

All participants were screened for MetS based on the criteria set by WHO [7], NCEP ATP III [10], and the IDF [11].

### 2.5. Data Analysis

Data was entered and analyzed using SPSS version 21. Results were presented as mean ± SD for normal variables and median (1st–3rd quartiles) for nonnormal variables. Statistical differences between metabolic syndrome and normal patients were tested using the Student *t*-test for normal variables and Mann–Whitney *U* test for nonnormal variables. Significance was set at *p* < 0.05.

## 3. Results

The clinical characteristics of all participants are outlined in Table 1. The mean (±SD) age was 42.4 (10.3) years for the MetS group and 33.1 (8.7) years for the non-MetS group. The mean (±SD) BMI was 33.1 (5.6) for the MetS group and 33.1 (8.7) for the non-MetS group. SPX levels were reduced in the MetS group (SPX = 0.18 ng/ml) compared to those in the non-MetS group (SPX = 0.26 ng/ml). Low levels of SPX showed an inverse relationship with high levels of fasting blood glucose (FBG = 6.2 ± 0.7, *p* < 0.001), systolic blood pressure (124.9 ± 14.6 mmHg, *p* < 0.001), diastolic blood pressure (78.2 ± 10.2 mmHg, *p* < 0.001), and triglycerides (1.8 mmol/l, *p* < 0.001) in the MetS group. Lower SPX levels were also associated with low HDL cholesterol (1.0 ± 0.3 mmol/l, *p* < 0.001) in the MetS group. Hemoglobin A1c (5.6 ± 0.6, *p* < 0.001) was significantly higher in the MetS group than in the non-MetS group as well as serum 25(OH)D. Other clinical parameters were measured, and there was no difference between the two groups (Table 1).

**Figure 1:**
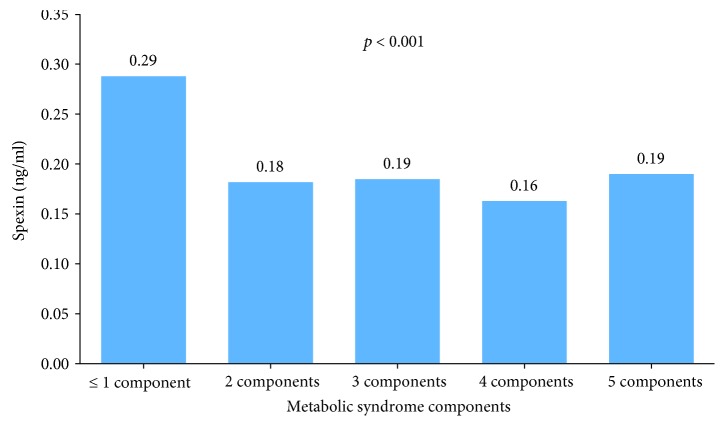
Median spexin (ng/ml) levels according to MetS components.

Median (IQR) SPX levels according to WHO, NCEP ATP III, and IDF MetS criteria were significantly lower in participants with MetS (0.18 ng/ml, *p* < 0.001) vs. non-MetS (0.27 ng/ml), MetS (0.18 ng/ml, *p* < 0.001) vs. non-MetS (0.26 ng/ml), and MetS (0.17 ng/ml, *p* = 0.009) vs. non-MetS (0.21 ng/ml), respectively. However, analyzing SPX levels by sex indicated that SPX levels were significantly lower only in female participants with MetS (Table 2). Furthermore, age and BMI did not show significant correlations with SPX levels. Finally, levels of SPX were observed to have a downward trend with respect to the presence of more than one MetS risk factor (Figure 1).

## 4. Discussion

In this study, we found significantly lower circulating levels of SPX in participants with MetS as compared to participants without MetS regardless of the definition used but more so using the WHO definition. The lower concentrations of SPX observed in this study are in agreement with that reported in severely obese adults [2]. SPX levels have been noted to be inversely correlated with fasting blood glucose in adults with type 2 diabetes [3], similar to the current study. In contrast, SPX did not show significant relationship with cardiometabolic risk factors in children (Kumar et al., 2017; Kumar et al., 2015). The differences between the result in our study and those conducted on children could be related to differences in physiology and the regulation of various metabolic factors in children vs. adults. Several recent studies have highlighted possible theories on the link between SPX and cardiometabolic indices. Sassek et al. [14] in their study observed that SPX may be strongly involved in the pathogenesis of diabetes or its recovery because of the effects of SPX on insulin secretion *in vitro* and *in vivo* and also on cell viability and proliferation. Lin et al. [15] highlighted the role of SPX in bile acid synthesis and reported correlations between serum SPX and total cholesterol in rats.

SPX as hormone has been shown to be involved in weight regulation with potential for obesity therapy as well as presence of gestational diabetes [4, 16]. SPX has been found to be expressed in brain regions and peripheral tissues of human, mouse, rat, and goldfish, including the hypothalamus, cerebral cortex, hippocampus, optic tectum, pons, retina, esophagus, stomach, kidney, liver, ovary, and adrenal glands [1, 3, 17, 18]. In addition, in pregnant women with gestational diabetes mellitus, SPX showed associations with immunological factor IL1-*β* and other cardiometabolic factors [19]. However, the exact mechanism of action of SPX is still unclear because of the lack of information on the SPX receptor [20]. Such genetic studies can provide additional information that can explain the link between SPX to innate immunity and insulin resistance, similar to the nonsense polymorphism found on the TLR5 gene that encodes innate immunity receptor, which was observed to protect from obesity predisposes to diabetes [21]. Evidences are however accumulating, and these suggest multiple physiological functions of SPX due to its expression in various tissues. The present study shows that the SPX level, regardless of the MetS definition used, is significantly lower in those without MetS. Furthermore, our study showed that SPX levels are inversely associated with MetS components and having more than one component at the same time results in a significant decrease in SPX levels compared to having less risk factors. Thus, we suggest that SPX levels should be regarded as a potential biomarker for MetS.

The authors acknowledge some limitations. Given the cross-sectional nature of the study, the causal relation of SPX to MetS cannot be determined. Furthermore, factors that might affect SPX such as dietary intake and physical activity were not taken into consideration. In addition, the results of this should be interpreted as caution because of the small and female-dominated sample size.

In conclusion, lower circulating SPX concentration in adults is associated with MetS classified under WHO definition even after adjustment for age and BMI. This association is also sex-specific, with MetS and SPX association apparently observed to be more significant in women. This observation must be explored further to clarify the role of SPX in the context of MetS. Further investigation and additional studies in larger populations are required to understand the exact function of this peptide and to validate the observations in the current study.

## Figures and Tables

**Table 1:** General characteristics of participants according to the presence of MetS.

Parameters	Non-MetS	MetS	*p* value	*p* value^∗^
*N*	136 (52.3)	124 (47.7)	—	—
M/F	21/115	41/83	—	—
Age (years)	33.1 ± 8.7	42.4 ± 10.3	<0.001	—
BMI (kg/m^2^)	27.6 ± 5.6	33.1 ± 5.6	<0.001	—
Waist-hip ratio	0.8 ± 0.1	0.9 ± 0.1	<0.001	<0.001
Systolic blood pressure (mmHg)	112.9 ± 13.3	124.9 ± 14.6	<0.001	<0.001
Diastolic blood pressure (mmHg)	68.1 ± 10.0	78.2 ± 10.2	<0.001	<0.001
Glucose (mmol/l)	5.1 ± 0.9	6.2 ± 0.7	<0.001	<0.001
Hba1c (%)	5.1 ± 0.6	5.6 ± 0.6	<0.001	<0.001
Triglycerides (mmol/l)^#^	1.2 (0.9–1.5)	1.8 (1.3–2.4)	<0.001	<0.001
Total cholesterol (mmol/l)	4.9 ± 1.3	5.0 ± 1.2	0.61	0.958
HDL cholesterol (mmol/l)	1.3 ± 0.4	1.0 ± 0.3	<0.001	<0.001
LDL cholesterol (mmol/l)	3.0 ± 1.0	3.1 ± 1.0	0.75	0.92
25(OH)D (nmol/l)^#^	34.8 (23.0–58.6)	39.5 (24.1–68.1)	0.04	0.90
Calcium (mmol/l)	2.3 ± 0.2	2.2 ± 0.2	0.71	0.39
Phosphorous (mmol/l)	1.1 ± 1.0	1.2 ± 0.3	0.46	0.49
Spexin (ng/ml)^#^	0.26 (0.17–0.5)	0.18 (0.13–0.2)	<0.001	0.33

Note: Data presented as mean ± SD and medians (1st quartile–3rd quartile) for nonnormal variables, respectively; # indicates nonnormal variables; ∗ indicates *p* values adjusted for age and BMI.

**Table 2:** Spexin levels according to various definitions of MetS.

	Median (*Q*1–*Q*3)	*p* value	*p* value^∗^
*MetS criteria (overall)*			
NCEP ATP III			
Non-MetS	0.26 (0.17–0.50)	<0.001	0.33
MetS	0.18 (0.13–0.24)		
World Health Organization (WHO)			
Non-MetS	0.27 (0.17–0.50)	<0.001	0.05
MetS	0.18 (0.13–0.23)		
International Diabetes Federation (IDF)			
Non-MetS	0.21 (0.16–0.50)	0.009	0.95
MetS	0.17 (0.13–0.31)		
*Female*			
NCEP ATP III			
Non-MetS	0.28 (0.18–0.58)	0.001	0.62
MetS	0.19 (0.15–0.33)		
World Health Organization (WHO)			
Non-MetS	0.31 (0.18–0.55)	<0.001	0.18
MetS	0.19 (0.13–0.31)		
International Diabetes Federation (IDF)			
Non-MetS	0.28 (0.18–0.58)	0.03	0.89
MetS	0.19 (0.15–0.37)		
*Male*			
NCEP ATP III			
Non-MetS	0.18 (0.14–0.20)	0.56	0.34
MetS	0.16 (0.13–0.19)		
World Health Organization (WHO)			
Non-MetS	0.17 (0.12–0.20)	0.87	0.42
MetS	0.16 (0.13–0.19)		
International Diabetes Federation (IDF)			
Non-MetS	0.16 (0.12–0.19)	0.96	0.80
MetS	0.16 (0.13–0.19)		

Note: Data presented as medians (1st quartile–3rd quartile). ∗ indicates *p* values adjusted for age and BMI.

## Data Availability

The data used to support the findings of this study are included within the article.
